# Patent conversion of a novel closed chest drainage device

**DOI:** 10.1186/s13019-024-02873-x

**Published:** 2024-07-10

**Authors:** Shaoqing Huang, Xu Song, Zhongkai Tong, Qiang Shi, Jie Li

**Affiliations:** 1 Department of Thoracic Surgery, Ningbo No.2 Hospital, 41 Xibei Road, Ningbo, 315010 China; 2 Department of Respiratory and Critical Care Medicine, Ningbo No.2 Hospital, 41 Xibei Road, Ningbo, 315010 China

**Keywords:** Closed chest drainage, Lung resection surgery, Negative pressure suction, 3D printing

## Abstract

Closed chest drainage is typically necessary following Lobar and Sublobar resections to evacuate gases and fluids from the thoracic cavity, eliminate residual pleural space for lung expansion, and maintain negative pressure. Currently, three conventional closed chest drainage systems are commonly employed: single-chamber, double-chamber, and triple-chamber systems; each system has its own advantages and disadvantages. Despite the emergence of digital drainage systems in recent years, their high cost hinders their widespread adoption. Based on this premise, our research team has achieved a patent for a micro air pump-integrated chest closed drainage bottle, which has been further developed into a novel device integrating a three-chamber system with negative pressure control and power supply capabilities. This device enables patients undergoing perioperative lung procedures to ambulate freely while simultaneously receiving chest suction therapy—a concept that theoretically promotes rapid postoperative recovery. Moreover, this device offers economic benefits and holds potential for clinical implementation (particularly in economically underdeveloped regions). In this article, we modified the thoracic closed drainage device based on our patent and presented this novel thoracic closed drainage device after 3D printing and assembly.

## Introduction

**Fig. 1:**
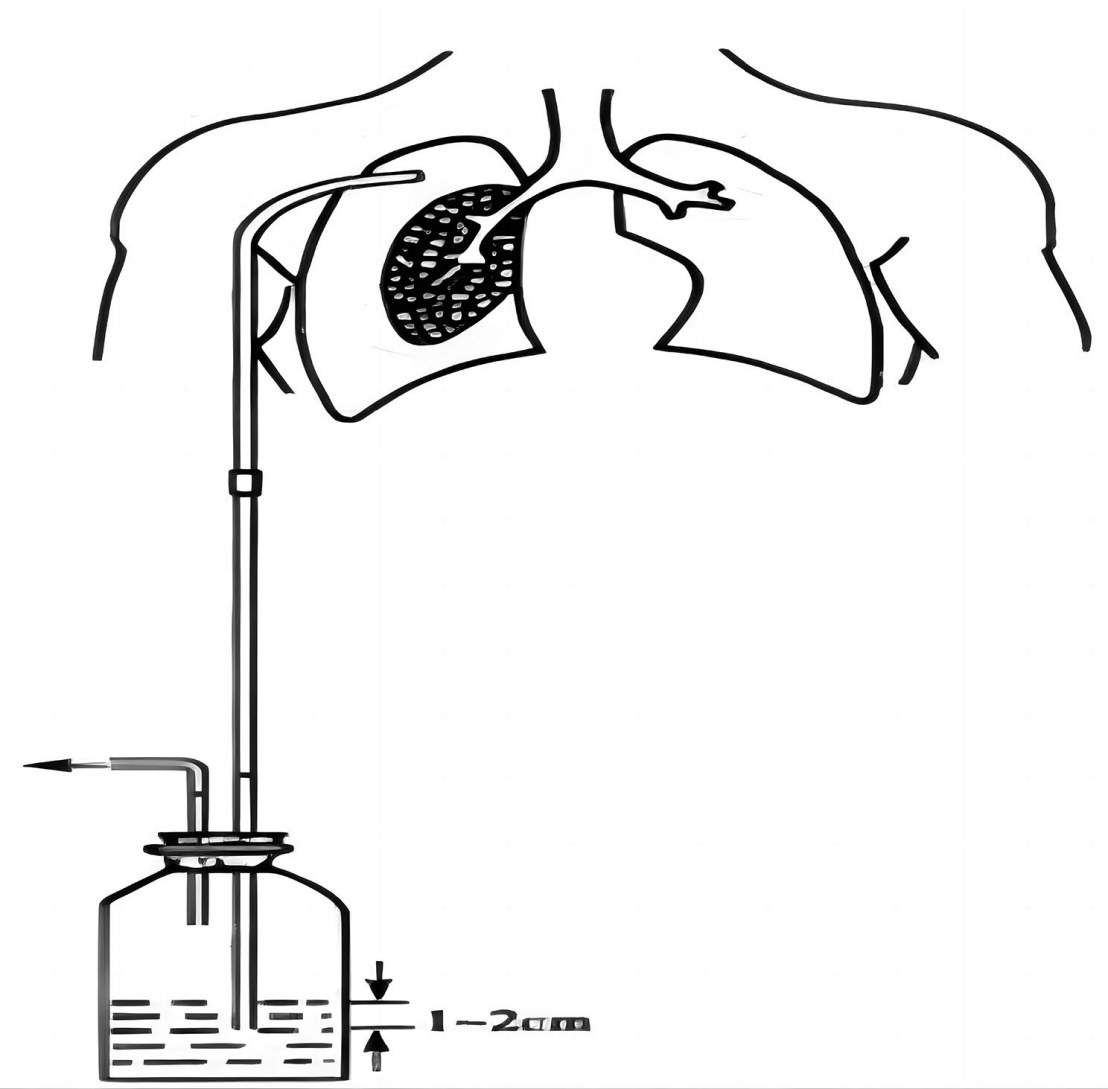
Closed chest drainage systems

**Fig. 2:**
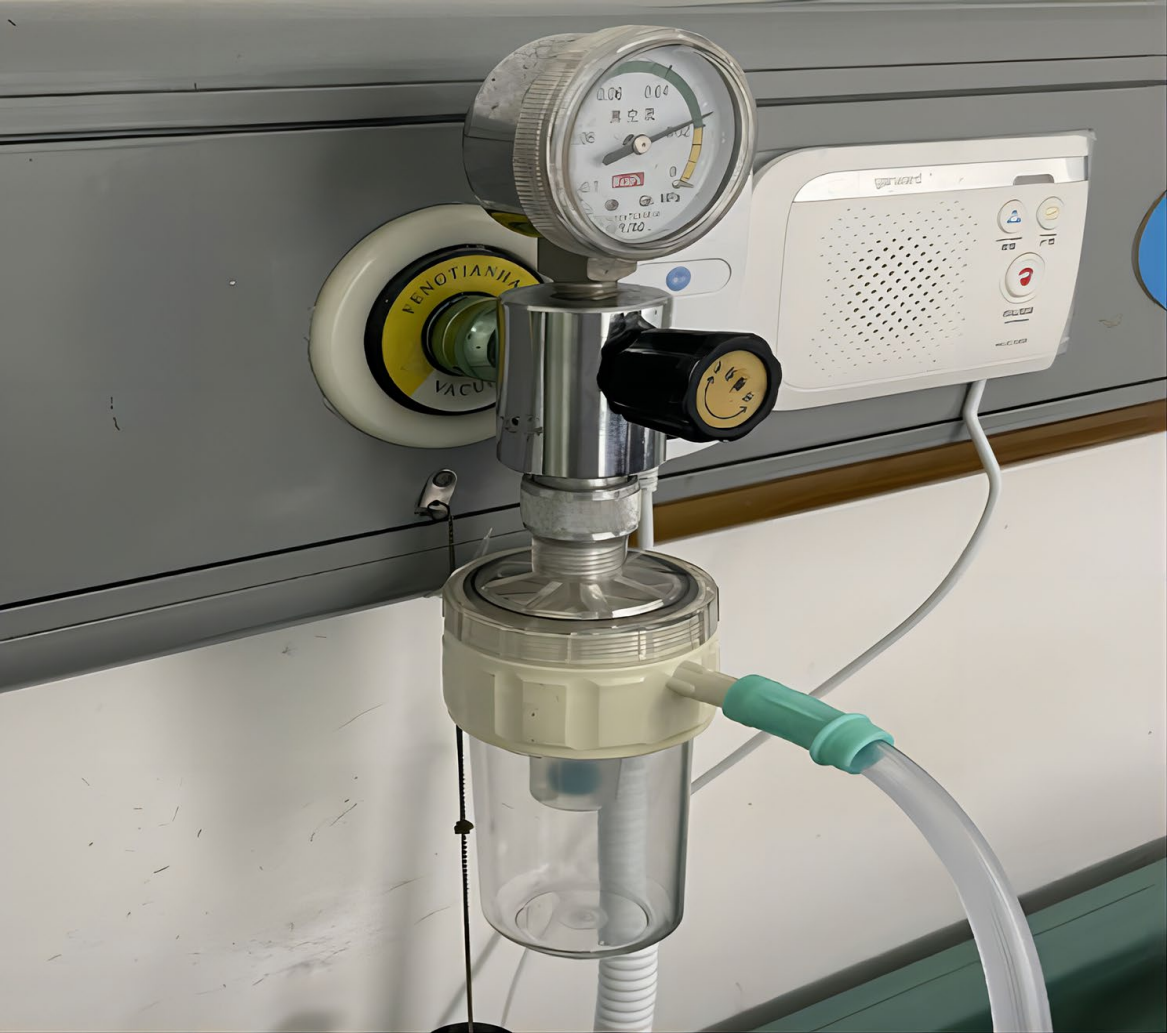
Wall type negative pressure

Lung cancer is the second most common type of cancer worldwide and the leading cause of cancer-related deaths [1]. Despite significant advancements in cancer management, anatomical resection remains the most effective approach for treating early-stage non-small cell lung cancer [2, 3]. Following lung resection surgery (Fig. 1), closed chest drainage systems are routinely employed [4]. There are two main approaches for managing closed chest drainage systems: simple water seal (nonsuction drainage) or water seal combined with external suction (suction drainage) methods [5]. However, there is ongoing controversy among clinicians regarding the optimal management strategy after lung resection surgery, particularly concerning the immediate implementation of suction drainage [6–9]. Undoubtedly, a suction drainage system facilitates faster clearance of air and fluid while promoting lung reinflation. Clinical studies have demonstrated that suction drainage reduces the duration of air leakage, shortens the duration of chest tube removal, and decreases the length of hospital stay without increasing complications [10–12]. Traditional suction drainage involves a wall-type negative pressure system (Fig. 2), which necessitates connecting negative pressure drain tubes to wall-mounted devices. This suction system hinders postoperative patient ambulation and may affect pulmonary function exercise as well as prevent lower extremity deep vein thrombosis. To address this issue, our study designed and developed an economically viable and portable suction-based closed chest drainage device.

### Closed chest drainage single-chamber system

The single-chamber system comprises a bottle connected to the chest drainage tube and a rigid chest tube submerged approximately 2 centimeters below the water surface, forming a unidirectional valve. This system facilitates the escape of air from the pleural cavity during respiration, effectively addressing pneumothorax. However, due to design limitations, several complications may arise: when the rigid suction tube is positioned above the water level, it loses its one-way valve function, leading to pneumothorax. As the fluid level in the bottle rises, increased pressure is required to expel additional air, resulting in elevated pleural cavity pressure and impeding unobstructed drainage. Furthermore, if the bottle is situated above the insertion point of the chest drainage tube, fluid reflux into the pleural cavity occurs, which exacerbates fluid accumulation while diminishing respiratory function and increasing pulmonary edema risk. Currently, due to these risks and the inability to enhance or adjust suction for draining chest fluid without compromising device effectiveness and safety measures, single-chamber systems are not commonly employed.

### Closed chest drainage two-chamber system

Lilienthal made advancements to the single-chamber closed chest drainage system in 1926, incorporating two chest vials for postpneumonectomy care of bronchiectasis. In this system, a chest drain tube is connected within the first bottle where chest fluid is collected, while the second bottle’s tube is positioned below a horizontal plane of 2 cm to create a unidirectional valve and facilitate air drainage from the thoracic cavity. A significant advantage of this dual-bottle system lies in its ability to enhance pleural space fluid removal without compromising drainage efficiency. However, similar risks associated with single-chamber systems persist due to the reliance on hydroseal placement and chest insertion site positioning. Furthermore, as it relies solely on gravity for facilitating drainage and lacks negative pressure control application, it may not provide sufficient efficacy for patients experiencing air leaks.

### Closed chest drainage three-chamber system

In 1952, Howe reported on a three-chamber chest drainage system comprising a collection bottle, a water seal bottle, and a negative pressure control bottle. This innovative system laid the foundation for modern chest drainage devices. The three-chamber device is connected in series with the chest drainage tube: the drainage chamber connects to the chest drainage tube, while the intermediate chamber functions as the water seal chamber (which must be maintained in an upright position for proper functioning). The third chamber controls negative pressure intensity through fluid level regulation using an external negative pressure system; in fact, the height of the fluid level determines the strength of the negative pressure, and the external negative pressure system determines the speed of the extraction of gas. This three-chamber configuration enables assessment of chest tube patency and confirmation of its placement within the pleural cavity. Air leaks can be identified by observing bubbles in the water seal chamber, and subjective quantification of leak severity (exhalation, inhalation, continuous or forced) allows for classification into severe, moderate to severe, moderate or mild categories. Notably, the utilization of external negative pressure, which enhances gas and fluid evacuation efficiency, is advantageous for facilitating lung re-expansion. However, it should be acknowledged that negative pressure drainage may induce patient discomfort due to alterations in pleural cavity pressure; moreover, noise generated from negative pressure could disturb patient restfulness. Additionally, traditional wall-mounted negative pressure systems might restrict patient mobility, impeding postoperative recovery.

## Method

**Fig. 3:**
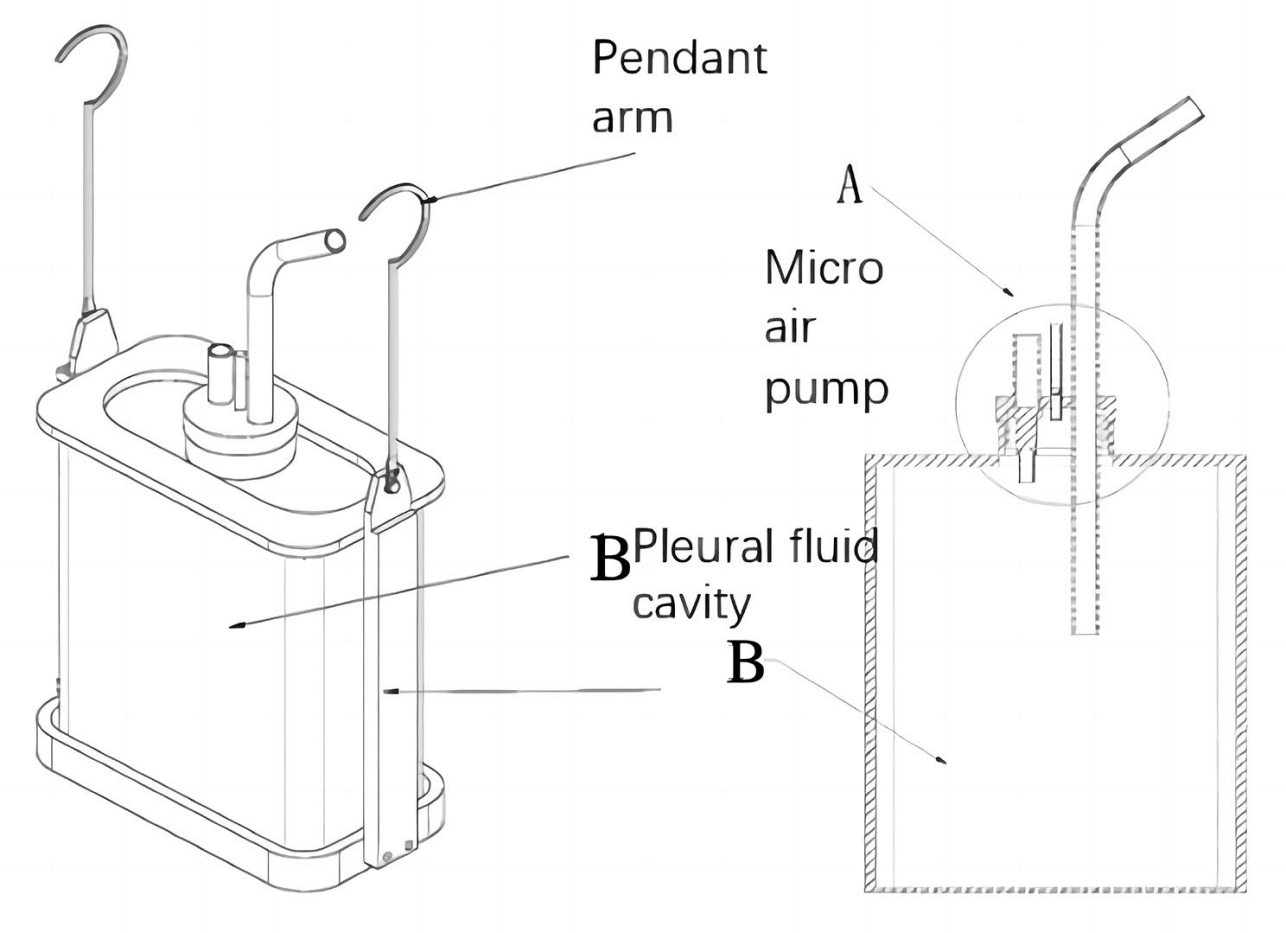
Closed chest drainage single-chamber system with a miniature air pump

We placed a miniature air pump at the entrance of the single-chamber system (Fig. 3) to extract gas from within the system and regulate internal pressure, partially addressing the aforementioned limitations of this setup. Additionally, in emergency patient rescue scenarios or departments with high patient volumes, reliance on wall-mounted negative pressure devices can be burdensome. This single-chamber system obviates the need for an accompanying negative pressure device, thus alleviating concerns associated with strained negative pressure resources to some extent. We have obtained a Chinese national patent for this innovation (patent number ZL 202122505281.4).

**Fig. 4:**
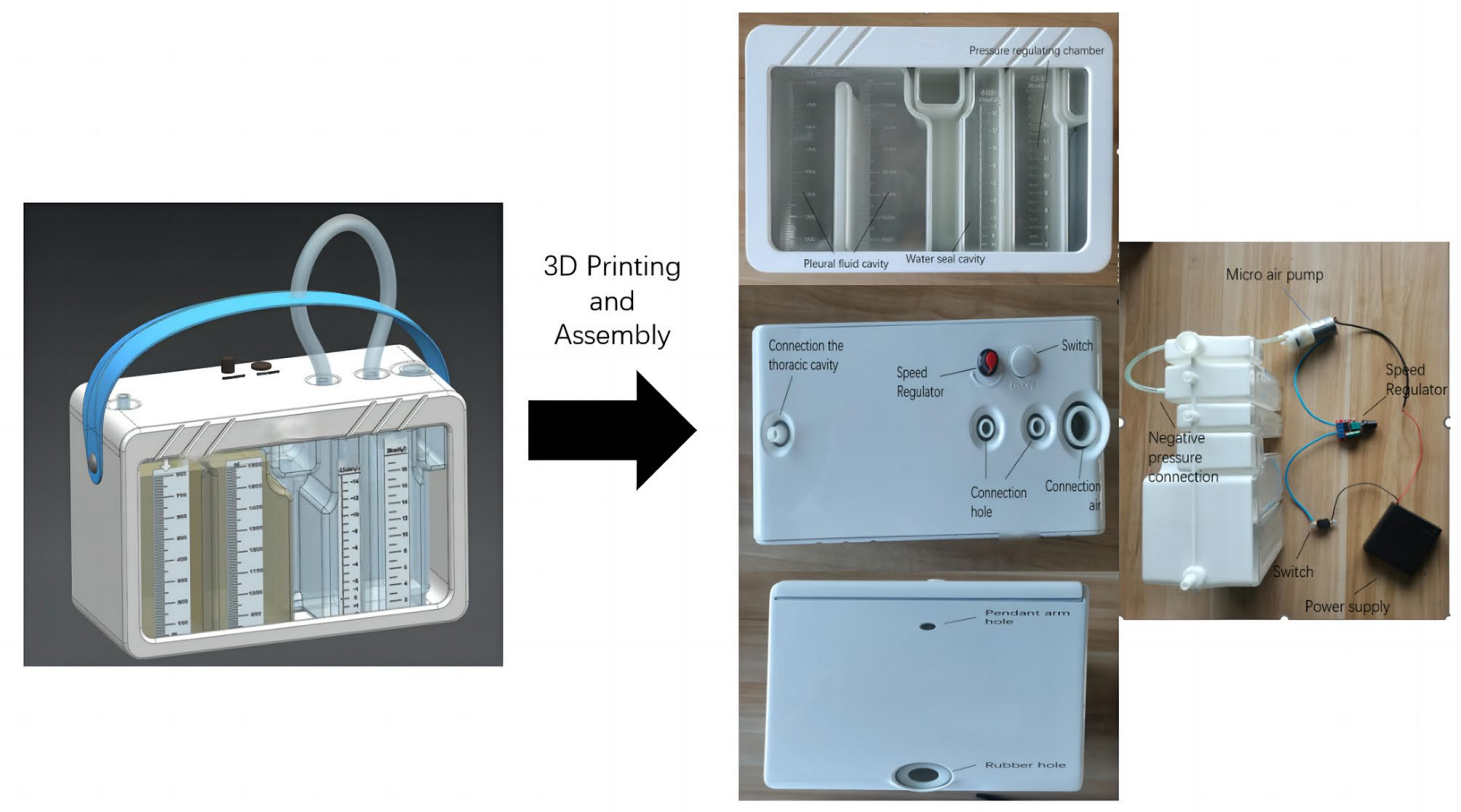
A novel closed chest drainage device

However, the aforementioned design does not adequately address the negative pressure and power control system of the closed-chest drainage device. Therefore, we further enhanced the negative pressure and power control system by implementing a three-chamber system and incorporating a rechargeable power supply based on our patent. The integrated device (Fig. 4) combines the three-chamber system, negative pressure control system, and power system into one unit, enabling patients to engage in postoperative activities following lung resection without compromising their functionality (the conventional negative pressure wall system restricts the movement of patients). In theory, this device is allowed to adjust the strength of the negative pressure. The height of the fluid level in the third chamber represents the suction strength. The micropump plays a role in pumping out the gas from the chamber, and the power of the micropump also determines the speed of pumping out the gas. The device currently has been 3D printed and assembled with a miniature air pump and rechargeable power supply. The maximum suction speed of the miniature air pump can reach 2.4 L/min. The capacity of the rechargeable power supply reaches 20,000 Ma. The device also includes a switch and a speed regulator. We made a 1 cm diameter rubber hole on the side of the device.

## Discussion

With the advancement of medical concepts and technology, closed chest drainage devices have undergone progressive enhancements from a single-room system to a double-room system and finally to a three-room system. However, there has been limited substantial progress in recent decades. Currently, the discourse on chest tube management primarily revolves around whether immediate application of negative pressure suction after lung resection is necessary and the optimal degree of negative pressure needed. Nevertheless, several studies advocate immediate postoperative negative pressure suction because it facilitates rapid patient recovery and reduces hospitalization duration. This viewpoint is in line with the 2017 translational medicine guidelines, which recommend the immediate application of negative pressure suction following lung resection. Clinically, we usually perform negative pressure thoracic closed drainage 24 to 48 h after lung surgery. However, conventional three-chamber closed chest drainage bottles require an external wall-type negative pressure source to achieve effective drainage, restricting postoperative patient mobility after lung resection. To address this limitation, we incorporated a micropump and rechargeable supply into a three-chamber drainage system to enhance the power supply and optimize control over negative pressure levels. By design, this is a wet system because a three-chamber closed drainage system is used instead of a mechanical negative pressure system. On the one hand, the expected advantages are as follows: (1) This system may be safer than a fully mechanical negative pressure system because even if our micropump failed, the three-chamber closed drainage bottle would still function as a complete thoracic closed drainage system. (2) The user-friendly one-key start and flow rate button located at the top of the device improve operational ease and user safety. (3) A rubber hole measuring 1 cm in diameter is strategically placed on the side of the device to facilitate convenient extraction of chest fluid samples for testing by medical personnel. (4) By using a rechargeable power supply (with a standard endurance of over 48 h), it is possible to charge the device using bedhead power sockets during patient rest periods, thus extending its operating duration without affecting functionality when patients move freely. (5) The cost is very low, at only approximately 20 US dollars, which provides an economical basis for clinical promotion. On the other hand, the novel device also has disadvantages: (1) The maximum negative pressure strength will be limited because the height of the liquid level in the three-chamber system is finite. (2) The weight of the device itself increases with the height of the liquid level, which is not beneficial to the patient. (3) The flow speed of air is limited by the power of the micropump, which may not be sufficient for large air leaks.

In summary, we modified the thoracic closed drainage device based on our patent and presented this novel chest closed drainage device after 3D printing and assembly. Furthermore, we discussed the potential advantages and disadvantages of this approach. In the next step, we will use this novel device in clinical trials.

## Data Availability

No datasets were generated or analysed during the current study.
